# Natural selection and local adaptation of blood pressure regulation and their perspectives on precision medicine in hypertension

**DOI:** 10.1186/s41065-019-0080-1

**Published:** 2019-01-07

**Authors:** Boon-Peng Hoh, Thuhairah Abdul Rahman, Khalid Yusoff

**Affiliations:** 1 Faculty of Medicine and Health Sciences, UCSI University, Cheras, 56000 Kuala Lumpur, Malaysia; 2 Chinese Academy of Sciences Key Laboratory of Computational Biology, Max Planck Independent Research Group on Population Genomics, CAS-MPG Partner Institute for Computational Biology, Shanghai Institutes for Biological Sciences, CAS, Shanghai, 200031 China; 3 Clinical Pathology Diagnostic Centre Research Laboratory, Faculty of Medicine, Universiti Teknologi MARA, Sungai Buloh Campus, 47000 Sungai Buloh, Selangor Malaysia

**Keywords:** Hypertension, Natural selection, Precision medicine

## Abstract

Prevalence of hypertension (HTN) varies substantially across different populations. HTN is not only common – affecting at least one third of the world’s adult population – but is also the most important driver for cardiovascular diseases. Yet up to a third of hypertensive patients are resistant to therapy, contributed by secondary hypertension but more commonly the hitherto inability to precisely predict response to specific antihypertensive agents. Population and individual genomics information could be useful in guiding the selection and predicting the response to treatment – an approach known as precision medicine. However this cannot be achieved without the knowledge of genetic variations that influence blood pressure (BP). A number of evolutionary factors including population demographics and forces of natural selection may be involved. This article explores some ideas on how natural selection influences BP regulation in ethnically and geographically diverse populations that could lead to them being susceptible to HTN. We explore how such evolutionary factors could impact the implementation of precision medicine in HTN. Finally, in order to ensure the success of precision medicine in HTN, we call for more initiatives to understand the genetic architecture within and between diverse populations with ancestry from different parts of the world, and to precisely classify the intermediate phenotypes of HTN.

**Electronic supplementary material:**

The online version of this article (10.1186/s41065-019-0080-1) contains supplementary material, which is available to authorized users.

## Introduction

Hypertension is a quantitative and multi-factorial trait. Hypertension (HTN) is defined as persistent elevation in BP greater than 140/90 mmHg [1]. It is either primary (essential) or secondary whereby the former is not an outcome of other disorders such as endocrinopathies (eg: Conn’s syndrome, Phaeochromocytoma, acromegaly) or renal pathologies (eg: chronic glomerulonephritis, renal artery stenosis) where it would be classified as secondary HTN. Both situations may have substantial interplay between genetic and environmental factors.

The prevalence of HTN is on a steady rise today; yet up to a third of hypertensive individuals do not achieve the target BP despite being on multiple anti-hypertensive medications [2]. Individuals with BP that is persistently above goals despite the use of three anti-hypertensives at optimum doses are categorized as having “drug-resistant hypertension (DRH)” [3]. Ethnic variations in BPs and differences in response to anti-hypertensive therapies though, have been well recognized [4, 5]. Numerous factors have been invoked to explain DRH: non-compliance to medications, age, gender, circadian rhythm, individual lifestyle and physical activity, the inability to prescribe an appropriate specific anti-hypertensive treatment due to the inability to predict the efficacy of the drug in a specific individual [6, 7], genetic background of an individual, and genetic variability between individuals [3, 8]. New drug development may address this issue, but it may not necessarily be individualized. Genetic basis of population and individual variation that may contribute to DRH could be the key and which would shape precision medicine and its future. However this cannot be achieved without knowing the genetic ‘fingerprints’ that are responsible for the development and maintenance of HTN in individuals with varied genetic make-up.

This article reviews the possible primordial cause(s) of HTN by revisiting the evolutionary process of BP regulation and the impact of natural selection and local adaptation of anatomical modern human (AMH) to the variability of BP. We also explore the possible impact afforded by genetic variability to precision medicine in HTN.

## Molecular and physiological mechanisms of BP regulation

**Fig. 1:**
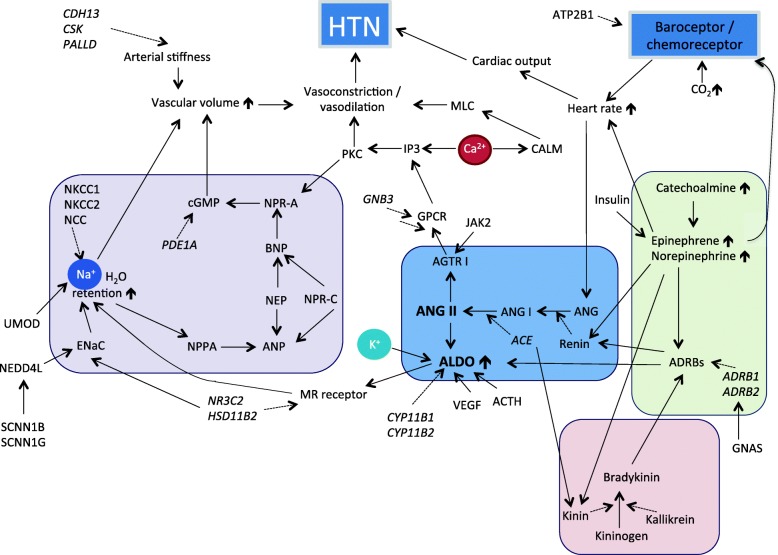
A simplified diagram of the molecular mechanisms of blood pressure regulation. The regulation of blood pressures involves complex interplay between (i) Kallikrein-Kinin system; (ii) Renal-Angiotensin-Aldosterone System (RAAS); (iii) natriuretic system; (iv) sympathetic nervous system; and (v) baroreceptor / chemoreceptor sensory

The mechanism of BP regulation has been well elaborated [9–12], particularly with respect to the model on renal mechanisms proposed by Guyton [13]. To summarize, it is a complex mechanism that involves integration of numerous biological systems in the brain, the kidneys, the blood vessels and the heart, which include, among others, the following principal mechanisms: (i) baroreceptor sensitivity on acute changes of BP in vessels; (ii) chemoreceptor responses to O_2_ and CO_2_ concentrations in the blood; (iii) natriuretic peptide of the brain and heart in response to elevated BP; (iv) the adrenergic receptor system and the sympathetic nervous system, which is responsible for heart rate and cardiac contraction; (v) Kinin-Kallikrein system that regulates vascular tone and renal salt homeostasis; (vi) Renin-Angiotensin-Aldosterone System (RAAS) that controls the vasoconstriction as well as salt homeostasis. Figure 1 illustrates an overall picture of the molecular and physiological BP regulation. The complex system of BP regulation involves at least 70 candidate genes; and many more identified via genome-wide association studies (GWAS), which impact on BP regulation via numerous various mechanisms (GWAS Catalogue: https://www.ebi.ac.uk/gwas/) (Additional file 1: Table S1) [14–16].

## Evolution and natural selection of BP in human survival

Natural selection is the key driving force for all evolutionary and adaptation processes. It is a process to improve the fitness of reproduction against environmental exposure by preserving the beneficial traits and discarding unfavourable ones. In other words, alleles that express traits with better adaptation capability will tend to increase in frequencies over generations and be preserved in a population; whilst alleles that express unfavourable traits decrease in frequencies. Nonetheless every trait under natural selection often has a trade-off – a negative trait may be favourable in another circumstance. A typical model is exemplified by the HbS mutation which causes sickle cell anaemia on one hand but is selectively advantageous against severe malaria infection on the other.

**Fig. 2:**
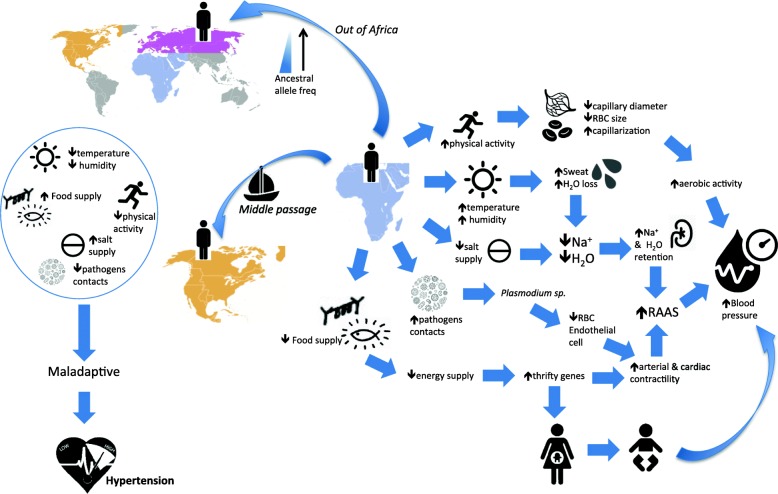
Illustration of the hypotheses of evolution and natural selection on the impact of blood pressure variability of the ancestors of anatomical modern human (AMH). During the Pleistocene Period, the ancient AMH practiced hunting-gathering lifestyle thus required higher physical stamina and endurance. Consequently an effective system of O_2_ exchange was evolved in such a way that smaller size of red blood cells, capillary diameters but denser capillarization to cater increasing sustained aerobic activity. As compensation to the physiological adaptation, systemic BP is elevated to suffice blood flow in the circulation. The savannah climate was hot and humid resulting in excessive sweat loss thus unavoidable sodium loss. The scarcity of salt further stringent deficiency. Kidney would then adapt an effective sodium and water retention which in turn elevated the renin-angiotensin-aldosterone system (RAAS) hence increase in blood pressure. Chronic infections with pathogens, notably the plasmodium sp. infection promotes disruption of RBC and effects on endothelial cells, subsequently triggered oxidative stress to the circulation, thus enhancing the renin-angiotensin-aldosterone system (RAAS) which in turn elevated BP. Low energy intake yet high requirement of physical stamina and endurance of the savannah hunters and gatherers often resulted in starvation. Prolonged starvation led to reduction of BP thus resulted in increased arterial tone and force of cardiac contraction to maintain BP and ensure organ perfusion. Therefore, genetic variation that enhances arterial and cardiac contractility, as well as those of energy-preservation preference was of selective advantage. In addition, foetus in the mother with low energy intake would alter the energy metabolism pathways to cope with famine. Postnatally in a nutrient rich environment, the altered physiological changes became maladaptive therefore increased risk of elevated BP. Out-of-Africa, natural selection and genetic drift may have resulted in lower ancestral allele frequencies of genes responsible for salt homeostasis in AMH habitation at higher latitude. Whilst during ‘middle passage’ and enslavement, a sudden drop of number of enslave caused a severe bottleneck. Those that survived however, are likely to carry excess frequencies of the ancestral alleles that allowed rapid adaptation to the extreme stress of dehydration. AMH carrying the ancestral alleles in modern lifestyle with resource enriched environment thus became maladaptive against the snail-speed evolution therefore increase risk of hypertension

Hypertension is thought to be a ‘by-product’ under the forces of natural selection and local adaptation [17, 18]. The evolution and natural selection of BP regulation in AMH is a process that involves continuous interaction between environmental changes and complex biological systems: the kidneys that regulate the homeostasis of sodium and water in the body, the respiratory systems for oxygen supply, and the cardiovascular system that ensures adequate circulation of blood and therefore O_2_ throughout the body to maintain vital functions [10, 11, 19]. It also involves various processes of metabolisms, as well as the mechanisms of body temperature regulation, which includes the ability to lose heat [12, 18, 20]. There are several hypotheses that can contribute to BP variability across populations (Fig. 2), which we shall elaborate. These processes could have occurred since the Pleistocene period prior to ancestral AMH migration ‘out-of-Africa’, presumably more than 200 thousand years ago. The ancient AMH lived in a savannah condition that was hot and humid [21]. Salt supply was extremely scarce. Indeed, the shortage of salt persisted until prior to the nineteenth century, where it was traded as a nutritional currency in Africa [22]. The ancestors of AMH were hunter-gatherers, which necessarily involved vigorous physical activity, yet they had very low total energy intake relative to their energy demands due to limited food availability.

(i) Increased systemic BP for a sustained physical performance:

The hunting-gathering lifestyle of the ancestors of AMH requires high physical stamina and endurance as they ran on an average of approximately 35 km in 2–5 h [20]. Essentially, smaller capillary diameters but denser capillarization increases the effectiveness of O_2_ exchange [19, 23], thereby increasing sustained aerobic activity which can maintain high physical performance. As compensation to the physiological adaptation, systemic BP is elevated to maintain sufficient blood flow in the circulation that could withstand the high capillary resistance generated from smaller capillary diameter. Whilst it has been proven true in cross-species studies [19], this hypothesis is also supported by the observation that athletes and physically active individuals generally have higher BP than those with sedentary lifestyles [24].

(ii) Heat adaptation, water and sodium retention:

Tropical climate and hunting-gathering lifestyle require effective heat dissipation via excessive sweat loss as proposed by previous studies which reported as much as 2 L/hour of sweat loss under these conditions [25, 26]. The resultant excessive sweat loss leads to unavoidable sodium loss and therefore disruption of fluid homeostasis that can affect muscle contraction, nerve impulse, glucose absorption and several other metabolic functions within the cells, which if persistent, could result in heart failure [18, 27, 28]. Given the critical importance of salt to human survival, natural selection could have been driven to prefer salt-avidity genetic variations, yet able to lose heat effectively [14]. In contrast, decreased blood volume due to fluid loss could exert increased stress on the cardiovascular system due to increased cardiac contractility to increase blood flow to the skin as a cooling down mechanism. Consequently, physical performance declined.

(iii) Energy metabolism and ‘Thrifty Gene’ hypotheses:

Low energy intake of the hunter-gatherers during Pleistocene period often led to extreme stress of starvation. During periods of prolonged starvation, BP drops in proportion to the significant reduction in available energy. In response, arterial tone and force of cardiac contraction increased in order to maintain BP and ensure organ perfusion [18, 26, 29]. Therefore, genetic variation that enhances arterial and cardiac contractility, as well as those of energy-preservation advantage, might have conferred survival advantage in the environmental context of early AMH evolution, which in turn led to increased BP as a counter balance.

In addition, it has been postulated that low energy intake and malnutrition in mothers could affect the embryo development and foetus health via altering or silencing gene expressions related to energy metabolism pathways in order to cope with famine, e.g. foetal cardiac gene programme switches the energy metabolism of the heart from fatty acid oxidation to glycolysis [30]. In modern lifestyle, the deprived adapted foetus lives in a nutrient rich environment postnatally, the physiological changes that were prevalent prenatally are detrimental, which in part could potentially increase the risk of elevated BP [31].

(iv) The Slavery Hypothesis:

The Slavery Hypothesis is one of the commonly accepted hypothesis that explains the high incidence of HTN and cardiovascular diseases (CVD) among the African Americans [32]. This hypothesis proposes that the African Americans possess increased risk of HTN and CVD as a consequence of strong selection during the period of ‘Middle Passage’ and enslavement. Historical records showed that large numbers of captive Africans who were shipped to the North America via the Atlantic Ocean died either during the journey, or soon upon arrival, due to poor living conditions which included dehydration, malnutrition, infectious diseases. The acute drop in numbers of enslaved Africans in North America resulted in a severe genetic bottleneck. Those that survived however, are likely to carry the ancestral variants (defined by allele that was carried by the common ancestor of the taxon, presumably chimpanzee as the outgroup species closest to human) with excess frequencies as a result of attempts to rapidly adapt to the extreme stress of dehydration.

(iv) Chronic infections:

Ancestors of AMH have been exposed to various infections, primarily attributed to their primitive life of hunting and gathering which exposed them to various pathogens. Several recent reports have attributed elevated BP to a number of infectious diseases, notably cytomegalovirus, periodontal bacteria and *plasmodium* sp. [33–37]. It was postulated that chronic exposure to these infections could have either triggered the pro-inflammatory cascade, or conferred oxidative stress to the circulation, thus enhancing the renin-angiotensin-aldosterone system (RAAS) which in turn elevated the BP [34].

Collectively, the genomic structure of ancestral AMH was shaped in a way that the ancestral alleles of the selected genes were better preserved and adapted to a resource-deficient environment. As the ancestors of AMH trekked across the savannah and inhabited other parts of the world, the snail-speed process of evolution could not cope with the rapidly changing climate and civilization. The selective advantage traits/variations, which in this case is elevated BP, has suddenly become a burden to the new lifestyles of improved hygiene conditions, enriched and increased food resources and sedentary lifestyle [17]. These hypotheses continued to be debated, with some claiming supportive evidence while others requiring further investigations. Herewith, we summarize several lines of findings that support these hypotheses.

From the epidemiological perspective, despite well recorded higher prevalence of HTN and CVD among the African Americans, numerous studies consistently noted that African populations (e.g Pygmy Bantu) have generally higher BP than the non-African populations [38]. Several other populations of African ancestry, such as the Haitian Black, the French Caribbean region of Martinique, French Guyana, and Guadeloupe [39, 40], or populations of phenotypically similar to the Africans (or often categorized as Negritos by anthropologists) such as the aboriginal populations from Australia, and indigenous Negrito populations from Peninsular Malaysia who are known to be hunter-gatherers, have been reported to have higher levels of BP than their counterparts in the non-indigenous population [41, 42]. This though intriguingly not seen among the hunter-gatherers from Europe [43]. Whilst general consensus to these observations is that socio-economy, lifestyles, education and awareness are the major contributing factors, the plausibility of population demographic and genetic architecture of these populations to BP elevation should not be disregarded.

**Fig. 3:**
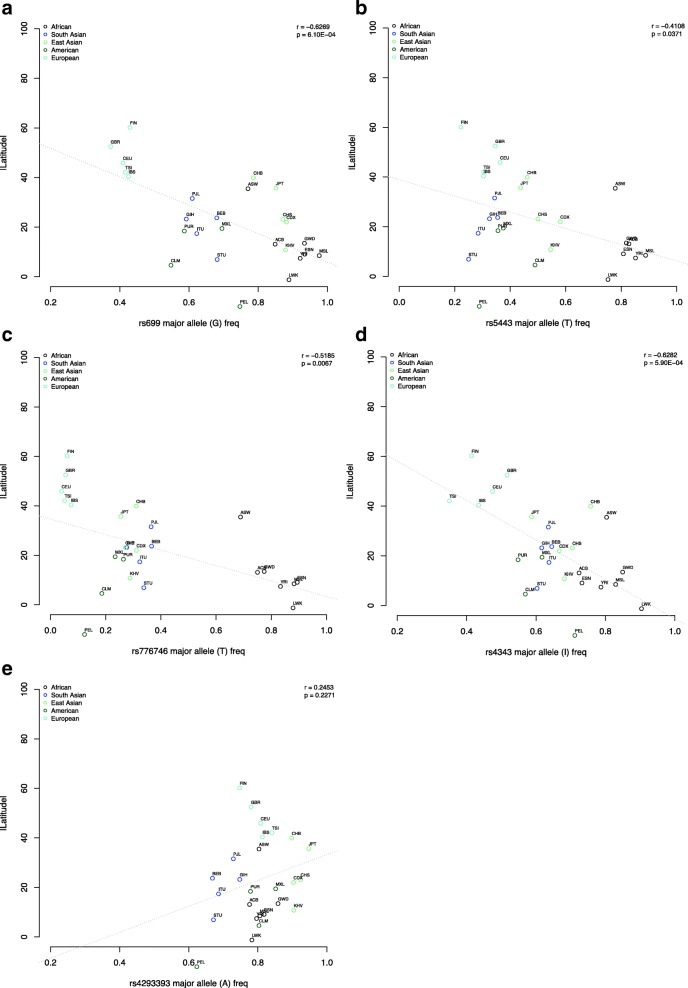
Correlation between the ancestral allele frequencies and latitude coordinates for the variants (**a**) rs699 (*AGT*), (**b**) rs5443 (*GNB3*), (**c**) rs776746 (*CYP3A5*), (**d**) rs4343 (*ACE*) and (**e**) rs4293393 (*UMOD*). Variant rs4343 is in strong linkage disequilibrium with the ACE I/D polymorphism. The ancestral allele A is linked with the insertion polymorphism. Significant correlations between the ancestral variant frequencies and the latitude coordinates are shown in all variants tested except rs4309 and rs4293393. However, distinct clusters are observed which indicates significant different ancestral allele frequencies between African, European, East Asian and American populations. ACB, African Carribean; ASW, African in Southwest US; BEB, Bengali in Bangladesh; CDX, Chinese Dai; CEU, European in Centre Utah; CHB, Han Chinese Beijing; CHS, Southern Han Chinese; CLM, Colombian; ESN, Esan Nigeria; Fin, Finnish, GBR, British; GIH, Gujurati Indian in Houston; GWD, Gambian; IBS, Iberian Spain; ITU, Indian Telugu in UK; JPT, Japanese Tokyo; KHV, Kinh Vietnam; LWK, Luhya Kenya; MSL, Mende in Sierra Leone Africa; MXL, Mexican in California; PEL, Peruvian; PJL, Punjabi Pakistan; PUR; Puerto Rican; STU, Sri Lankan; TSI, Toscani Italy; YRI, Yoruba Nigeria

From the population and evolutionary genetics perspective, a handful of candidate genes that are responsible in regulating water and salt retention have shown strong signals of natural selection, in particular between African ancestry and out-of-Africa populations. Notably, the derived alleles frequencies for variants rs699 of *AGT* have been repeatedly reported to correlate with geographical latitude coordinate [25, 44, 45] (Fig. 3); whilst *ACE* I/D (rs4343) variant has shown a deep coalescence time thus supporting balancing selection [46]. Other candidate genes involved in regulation of sodium reabsorption namely *GNB3* and *CYP3A* genes also show a similar trend pattern [25, 47].

HTN has been linked to malarial infection via several indirect mechanisms including malnutrition of pregnant mothers and foetus, as well as chronic inflammation. One significant supporting example is, females in regions with malaria hyper-endemicity are likely to have acquired immunity to prevent most febrile episodes, thus suffer impaired uteroplancental blood flow and maternal anaemia, which leads to foetal growth restriction subsequently low birth weight [34, 48]. Febrile malaria episodes that are more likely among women with low immunity are thought to induce uterine contractions, which are mediated by elevated levels of tumour necrosis factor-α (TNF α) leading to preterm birth [34, 49]; whilst Gallego-Delgado and Rodriquez provided supporting information that angiotensin II (Ang II) may protect against cerebral malaria, possibly via its effects on host vascular endothelial cells [50]. A candidate gene *CDH13* that was associated with HTN in a handful of GWAS [51–54], interestingly noted that it was also found to be significantly associated with susceptibility to malarial infection in a meta-GWAS analysis [55, 56]. Recent search revealed *CDH13* was one of the most prominent signals for positive natural selection in the Negrito populations from Peninsular Malaysia [57, 58]. Malaria threat among the Negritos has long been recorded [59, 60]; at the same time, more than half of this population are hypertensive [41]. Given the phenotypic observation and the fundamental role of this gene, it is plausible to postulate *CDH13* as a target of natural selection for malaria and HTN. However, we acknowledge that a direct correlation between malaria infection and blood pressure alteration is yet to be conclusively established, thus providing an insight on the direction of the selection pressure for this gene. In contrast, infection with cytomegalovirus enhances renin and Ang II activities, which in turn triggers RAAS, subsequently elevating BP [33, 61], may imply the complexity of the biology of BP regulation and infectious disease.

## The implications of evolutionary genetics to pharmacogenomics and precision medicine in HTN

Precision medicine refers to an approach that customizes healthcare tailored specifically to an individual patient or a particular group of patients [62]. Whilst numerous ‘-omics’ technologies such as the proteomics, metabolomics, lipidomics, metagenomics, epigenomics, microbiomes and genomics, have been proposed as a candidate solution for precision medicine, each has its own limitations. Some require sophisticated laboratory setup and are labour intensive, while other methods may not be sustainable or practical across time, or results are difficult to interpret. Pharmacogenomics is thought to be a favourite approach to the implementation of precision medicine because its findings, if validated, can be translated conveniently to bedside applications, hence impacting human health more rapidly than other ‘-omics’ technologies. Using such genetic-guided approach, clinicians could presumably be able to predict the response of a hypertensive patient to a selected medication, thus appropriate type(s) of medication(s) with an optimal dose could be definitively found for the patients. One such example is trastuzumab (Herceptin) treatment for breast cancer patients with human epidermal growth factor receptor (*HER2*, also known as *ERRB2*) mutations. *HER2* amplification is seen in 15–20% of the breast cancer patients, and is associated with faster growth rate and poorer diagnosis. [63, 64]. The use of trastuzumab is approved and recommended by FDA, when the breast cancer patients either have a *HER2* protein overexpression or *HER2* gene amplification. Nonetheless the key to the success of this implementation relies on two major determining factors:

(i) Identifying and prioritizing HTN associated causal variants between and within populations:
  We acknowledge that the genetic landscape of HTN is largely influenced by evolutionary forces such as natural selection and local adaptation. Ideally, candidate gene variant(s) under selective pressure are often functionally important to BP regulation. Surprisingly several variants that were functionally validated including rs699/rs5051 (*AGT* gene); rs5443 (*GNB3* gene) and rs4293393 (*UMOD* gene), which were thought to be suitable candidates, however, yielded contrasting results in association with BP in different populations [8, 15, 25, 65]. One postulation is that selection process of these functional variants could have taken place long time ago, and has reached a fixation stage across populations [66].
  However, a recent search on GWAS catalogue (https://www.ebi.ac.uk/gwas/) (using keywords ‘*blood pressure*’ and ‘*hypertension*’; date 11th February 2018) revealed as many as 457 GWAS signals that reached the genome-wide significant threshold at *p* < 10^− 7^, of which 283 were distinct, corresponding to 245 candidate genes (Additional file 1: Table S1). Moreover, only 7 candidate genes with established pathophysiological mechanisms of HTN were replicated in GWAS (*ACE1*, *ACE2*, *ADRB1*, *ADRB2*, *MME, CACNA2D2* and *UMOD*). These observations imply that (i) the genetic variants underlying HTN are relatively common (occurring > 1% in a population), have undergone little or no selection in earlier populations, and mutations (derived alleles) could have occurred before or during early AMH migration out-of-Africa; (ii) the physiology of HTN is probably more complex and heterogeneous than our current understanding;(iii) it remains a challenge to identify which genetic marker (or array of genetic markers) best suits the pharmacogenomics approach in HTN.
  Some encouraging progress has been seen albeit at a preliminary stage. For instance, a clinical trial was carried out to predict the investigating the response of diuretics on HTN patients who carried *UMOD* rs13333226-AA genotype [62] found that HTN individuals with the said genotype had increased UMOD excretion, thus greater salt sensitivity [67–69]. According to Dominiczak et al. (2017), a prospective *UMOD* rs13333226-AA genotype directed trial of a long-acting loop diuretic, torasemide, in uncontrolled hypertensive is currently ongoing [62]. Whereas two clinical trials, namely the Nordic Diltiazem (NORDIL) and Pharmacogenomic Evaluation of Antihypertensive Responses (PEAR) studies, applied the *NEDD4L* rs4149601 genotype to predict the response of HTN patients on thiazide diuretics [70, 71]. The rs4149601 G-variant creates a cryptic splice site in *NEDD4L*, which leads to less ENaC downregulation, thereby increased sodium retention. The NORDIL study claimed that the HTN individuals carrying the G allele showed better response to the thiazide/β-blocker treatment compared to those with AA genotype [71]; whereas in PEAR study, the rs4149601 G allele showed better response to hydrochlorothiazide but no difference by genotype in response to the β-blocker atenolol [72].
  The solution to this challenge however, relies on understanding the genetic architecture within and between a wide range of populations with ancestry from different parts of the world [73, 74]. In other words, cataloguing genomic variations that cover diverse populations, especially the indigenous populations are crucial to ensure the success of personalized medicine.
(ii) Identifying the intermediate phenotypes of HTN:
  The findings from GWAS imply that the pathophysiology of HTN is heterogeneous, thus in agreement with the postulations that multiple causative factors which interplay with different environment factors could have contributed to the elevation of BP in different populations. Therefore characterizing different intermediate phenotypes of HTN is crucial [7, 75–77]. Korner (2010) defined two major intermediate phenotypes of HTN – stress and salt-sensitive HTN and obesity related HTN, which could be further characterized by neural and non-neural mechanisms [75]. In addition, it was recognized earlier that there are two distinct physiological salt-sensitive HTN namely, the non-modulating salt-sensitive and the low renin salt sensitive [6]. Another intermediate phenotype of HTN has been recognized as the neurogenic HTN namely the deoxycorticosterone acetate (DOCA) salt hypertension, which exhibits salt-dependent of excess mineralocorticoid [12, 78].

Disease heterogeneity is the major source of variability in response to anti-HTN medication [79]. Precise ‘phenotyping’ therefore, is the key to identifying the causative gene(s) or variant(s) of HTN thus warrant the long-term success of pharmacogenomics and precision medicine. Several evolutionary drivers elaborated in this article may be able to shed lights to attempts of identifying different intermediate phenotypes for HTN. However, more efforts are required to comprehensively understand the physiology of HTN to categorize the disease phenotypes.

The concept of *precision medicine* may seem far-fetched at present. But looking back when the idea of going to the moon seemed impossible, man had taken this figment of imagination and realized it. The development of science and technology, particularly in the area of pharmacogenomics, has pointed us in the right direction though the journey remains long before we can even begin to consider personalized medicine. However, with future research advances, one day, precision medicine will no longer be a notion, but a reality and a way forward in medicine. But this can only be achieved with a comprehensive understanding on the complex ‘genotype-phenotype’ interactions.

## Concluding thoughts

Forces of evolution and natural selection could have resulted in variability in BP across global populations with diverse ancestry, which could have possibly led to different pathophysiological mechanisms or intermediate phenotypes of HTN. This in part resulted in different responses towards anti-HTN medications today. Whilst the Life Sciences community believes that the advancement of the genome sequencing and ‘-omics’ technology could provide a solution to the precision medicine in HTN, equally important is the identification of intermediate phenotypes of HTN (and its related biochemical traits); and the crucial cataloguing of both rare and common variants of various populations as reference to better understand the evolution of common traits like HTN, and how these variants found in different parts of the world influence HTN and its response towards medications.

## Additional file

Additional file 1: **Table S1.** List of candidate genes associated with blood pressure regulation. (DOCX 105 kb)

## Abbreviations

AMH: Anatomical modern human
Ang II: Angiotensin II
BP: Blood pressure
CVD: Cardiovascular disease
DRH: Drug-resistant hypertension
GWAS: Genome-wide association study
HTN: Hypertension
RAAS: Renin-Angiotensin-Aldosterone System
