# Supplementary material for: Natural selection and local adaptation of blood pressure regulation and their perspectives on precision medicine in hypertension
Source: Hereditas. 2019 Jan 7;156:1. doi: 10.1186/s41065-019-0080-1 (PMC6323824; doi:10.1186/s41065-019-0080-1)
Supplement: Supplementary file 1 — Table S1. List of candidate genes associated with blood pressure regulation. (DOCX 105 kb) [file 41065_2019_80_MOESM1_ESM.docx]

**Table S1. List of candidate genes associated with blood pressure regulation**

[1–7]

| **Candidate Genes** | **Remark** |
| --- | --- |
|  | **Renal Aldosterone Angiotensin System (RAAS):** |
| *AGT****^†^*** |  |
| *ACE1****^†^*** |  |
| *ACE2****^†^*** |  |
| *AGTR1* |  |
| *AGTR2* |  |
| *REN* |  |
| *CYP11B2* |  |
| *CYP11B1* |  |
| *NR3C2* |  |
| *CYP17A1†* |  |
| *HSD11B2 (11-β-hydroxysteroid dehydrogenase)* |  |
|  | **Na^+^ regulation and reabsorption related pathways:** |
| *CYP3A4* |  |
| *CYP3A5* |  |
| *ABCB1* |  |
| *ANP* |  |
| *BNP* |  |
| *NHE3* |  |
| *SGLT* |  |
| *SCNN1A (ENaC)* |  |
| *SCNN1B* |  |
| *SCNN1C* |  |
| *ATPA1* |  |
| *ATPA2* |  |
| *ATPA3* |  |
| *KCNJ1* |  |
| *KCNJ5†* |  |
| *WNK1* |  |
| *WNK4* |  |
| *CUL3* |  |
| *KLHL3* |  |
| *SLC12A1* |  |
| *SCL12A3* |  |
| *UMOD****^†^*** |  |
| *CORIN* |  |
| *SGK1* |  |
| *NEDD4L* |  |
| *CLCNKA* |  |
| *CLCNKB* |  |
| *ADD1* |  |
| *ADD2* |  |
| *ADM* |  |
| *ADM2* |  |
| *DRD1* |  |
| **Kallikrein-Kinin System:** |  |
| *BDKRB1 (Bradykinin 1)* |  |
| *BDKRB2 (Bradykinin 2)* |  |
| *ET1* |  |
| *ET2* |  |
| *ET3* |  |
| *ETAR* |  |
| *ETBR* |  |
| *ECE1 (Endothelin Converyting Enzyme 1)* |  |
| *CPM* |  |
| *CPN1* |  |
| *CPN2* |  |
| *KLK1* |  |
| *KLKB1* |  |
| *KNG1* |  |
| *MME (metalloendopeptidase/neutral endopeptidase)* |  |
| *SERPINA4* |  |
|  | **Calmodulin / Calceneurin / Calcium channel:** |
| *CALM1* |  |
| *CALM2* |  |
| *CAMK4****^†^*** |  |
| *CACNA2D2****^†^*** |  |
| *CACNA1D* |  |
| *ATP2B1* |  |
|  | **Sympathetic and parasympathetic nervous systems:** |
| *ADRB2* |  |
| *ADRB1****^†^*** |  |
| *GNAS* |  |
| *GRK4* |  |
| TH (tyrosine hydroxylase) |  |
|  | **Others:** |
| *PDE5A****^†^*** |  |
| *PDE3A* |  |
| *JAG1* |  |
| *ADAMTS16* |  |
|  | **GWAS signal: Significant threshold P <10^-7^** |
| *IGSF5* |  |
| *COL21A1* |  |
| *LOC107983984 - LOC105370464* |  |
| *PPP4R1L* |  |
| *MYB* |  |
| *CDH13* |  |
| *ARMC8P1 - TCEA1P3* |  |
| *HHAT* |  |
| *RNU2-8P - SLC25A5P7* |  |
| *LOC107985095 - LOC105378874* |  |
| *LINC01191 - SEPHS1P7* |  |
| *CAPZA1* |  |
| *PLEKHG1* |  |
| *CSMD1* |  |
| *CARM1P1* |  |
| *RASGRF2* |  |
| *LINC01102* |  |
| *LOC107984543 - BRWD1P2* |  |
| *UBA52P4 - LOC105377005* |  |
| *TRHDE* |  |
| *LOC283278* |  |
| *GSE1* |  |
| *C5orf56* |  |
| *LOC105378492* |  |
| *LINC01601 - LOC105376868* |  |
| *BMPR1B* |  |
| *LOC101927701 - LOC402076* |  |
| *LOC105378027* |  |
| *LOC101929528 - UBXN2B* |  |
| *LOC102724874 - LOC105375911* |  |
| *PLCXD3 - TCP1P2* |  |
| *CLNK* |  |
| *LOC105377882* |  |
| *PLEKHA7* |  |
| *THRB* |  |
| *LOC105373744 - LOC100131562* |  |
| *PAM* |  |
| *SLC5A5* |  |
| *DAPK1* |  |
| *DHFRP2* |  |
| *UVSSA* |  |
| *LOC107986943* |  |
| *LOC107986055 - SENP2* |  |
| *LOC107986178* |  |
| *PML - DNM1P33* |  |
| *NR2F2-AS1* |  |
| *UBE3AP2 - TIAM1* |  |
| *LOC105377369 - CCDC34P1* |  |
| *LOC107987026 - MTAP* |  |
| *TTC6* |  |
| *ANK1* |  |
| *OVCH2* |  |
| *FGF14* |  |
| *SERPINB7 - SERPINB2* |  |
| *RAB31* |  |
| *TENM4 - LOC105369405* |  |
| *UGP2* |  |
| *ITPR1* |  |
| *DPY19L2P1 - TBX20* |  |
| *BAG6* |  |
| *ZC3HC1* |  |
| *LOC102723576* |  |
| *MYO1D* |  |
| *OPRM1* |  |
| *LOC105377468 - LOC105377469* |  |
| *ENPP7* |  |
| *PBRM1* |  |
| *GPATCH2L* |  |
| *CASZ1* |  |
| *CLEC12B, LOC102724020* |  |
| *ARMC10P1 - LOC105373992* |  |
| *ST6GAL1* |  |
| *MARK2* |  |
| *LOC105374189* |  |
| *LOC107985518 - LOC107985519* |  |
| *LOC101927238 - SPRY2* |  |
| *CSK* |  |
| *LOC102723446* |  |
| *ZNF746 - TRC-GCA16-1* |  |
| *LINC01471* |  |
| *FGD5* |  |
| *MSRA* |  |
| *KDM4A* |  |
| *PARVB* |  |
| *LOC105377436* |  |
| *OVOS2 - LOC107987168* |  |
| *PODXL* |  |
| *CNTN4* |  |
| *LOC107984437 - LOC102723639* |  |
| *ABCB11* |  |
| *TUSC7 - MIR4447* |  |
| *LOC100419366 - LOC105370803* |  |
| *OR5H1 - LOC105373996* |  |
| *AMN1* |  |
| *NOBOX - RNU6ATAC40P* |  |
| *COLEC10 - LOC105375725* |  |
| *ALG1L* |  |
| *LOC101927605* |  |
| *WLS - RPS7P4* |  |
| *MAP2K4 - LINC00670* |  |
| *LOC107985950* |  |
| *C17orf112 - LOC645163* |  |
| *TMEM255B* |  |
| *RPL6P5 - LOC105373664* |  |
| *KIAA0040* |  |
| *HIVEP2* |  |
| *RABL2A* |  |
| *OBFC1* |  |
| *LOC101927078 - TRIM36* |  |
| *NUCB2* |  |
| *TBX2* |  |
| *LOC107984724 - ALDH1A2* |  |
| *SOX6* |  |
| *SAYSD1 - KCNK5* |  |
| *PKD1L1* |  |
| *NCKAP5 - LOC105373627* |  |
| *LOC105375896* |  |
| *IFT43 - GPATCH2L* |  |
| *LOC101927845* |  |
| *FAM185A, FBXL13* |  |
| *ARSJ* |  |
| *PTCD3* |  |
| *NPR3* |  |
| *FOXP2* |  |
| *LOC105372132 - LOC105372135* |  |
| *LOC339298* |  |
| *C1GALT1* |  |
| *LOC105369838 - LOC101928137* |  |
| *NPR3 - LOC340113* |  |
| *GPR20* |  |
| *CTNNA3 - AKR1B10P1* |  |
| *RBPMS2 - PIF1* |  |
| *HLA-DRB1* |  |
| *NCR3 - UQCRHP1* |  |
| *LPPR5* |  |
| *SH3TC2* |  |
| *HCG22 - C6orf15* |  |
| *RPL18AP17 - RPL21P110* |  |
| *ZNF318* |  |
| *INSR* |  |
| *SNTB1 - LOC100133147* |  |
| *LOC101929750* |  |
| *LOC105378769 - LINC00466* |  |
| *ZNF831* |  |
| *MIR5007 - HNF4GP1* |  |
| *TENM3* |  |
| *LOC102723323 - LOC107984892* |  |
| *ALX4 - LOC105376645* |  |
| *LOC105377865 - LOC107986613* |  |
| *IGL* |  |
| *PRPS1P1 - CYP2C56P* |  |
| *HIST1H2APS2 - SLC17A2* |  |
| *FRY* |  |
| *LINC00363 - GPC6* |  |
| *LOC107986335* |  |
| *PRKCA - CACNG5* |  |
| *CARS2* |  |
| *ARHGAP42* |  |
| *ULK4* |  |
| *TARID* |  |
| *RPL35P4 - LOC107986733* |  |
| *UGCG - RNU6-710P* |  |
| *LOC105369408 - RNU6-544P* |  |
| *LOC105374715 - RPS8P8* |  |
| *LOC105369684* |  |
| *STRCP1* |  |
| *MAP4* |  |
| *LRRC10B* |  |
| *GRAMD1B* |  |
| *RD3 - LOC107985261* |  |
| *GUCY1A3* |  |
| *TAF1C* |  |
| *HOXA3* |  |
| *NT5C2* |  |
| *LOC105373531 - C2orf40* |  |
| *RPS20P12 - LOC105373934* |  |
| *SLIT1* |  |
| *SRRM1 - CLIC4* |  |
| *MIR99AHG - RNU1-98P* |  |
| *RGS7* |  |
| *LOC105375278 - LOC107986738* |  |
| *LOC107986223* |  |
| *LOC105373781 - LOC107985832* |  |
| *LOC107984525* |  |
| *ALK* |  |
| *C20orf187* |  |
| *NAGLU* |  |
| *PDXK* |  |
| *BANK1 - SLC39A8* |  |
| *CLVS2* |  |
| *LYN* |  |
| *LOC101927181 - GRIFIN* |  |
| *CHD3* |  |
| *AGAP1* |  |
| *RGS21 - LOC105371664* |  |
| *LOC105378314 - MRPS35P3* |  |
| *LOC105369501 - LOC107984390* |  |
| *VAT1L - CLEC3A* |  |
| *KCNMA1* |  |
| *TTYH2* |  |
| *LOC388780 - TGM3* |  |
| *LOC100533736 - PRKRIRP9* |  |
| *CDH18* |  |
| *EBF2* |  |
| *KDM5A* |  |
| *ZNF385B* |  |
| *LOC105373223 - LOC107983951* |  |
| *SGCZ* |  |
| *PLCE1* |  |
| *LOC102723639 - LOC105370003* |  |
| *RNA5SP23 - PTGFR* |  |
| *IGSF5 - PCP4* |  |
| *FHIT* |  |
| *LOC100506532* |  |
| *SIPA1L2* |  |
| *RPS6KA2* |  |
| *ADARB2* |  |
| *GRB10* |  |
| *STK3* |  |
| *LOC105375230 - LOC105375231* |  |
| *DCBLD1* |  |
| *SNRPGP2 - DYNAP* |  |
| *LOC105377442 - PCDH18* |  |
| *AKT2* |  |
| *HSPB7* |  |
| *LOC107986913 - LOC105379224* |  |
| *FES* |  |
| *CACNB2* |  |
| *MYO16* |  |
| *MSL3P1 - TRPM8* |  |
| *LOC730129 - LOC102724419* |  |
| *OR51H1 - OR51H2P* |  |
| *LOC105377462* |  |
| *LOC105373833 - SPATS2L* |  |
| *NFKBIA* |  |
| *LOC105377871* |  |
| *LOC105377786, LOC105377787* |  |
| *PDE3A* |  |
| *LOC105372191 - FBXO15* |  |
| *EHBP1L1* |  |
| *NXN* |  |
| *SULT1C3 - WASF1P1* |  |
| *SYNJ2BP, SYNJ2BP-COX16* |  |
| *LOC101928111 - LOC401040* |  |
| *PRKG2* |  |
| *SLC39A8* |  |
| *LOC105379111 - RAB9BP1* |  |
| *KLHL32* |  |
| *LOC105378117* |  |
| *LOC105370726 - LOC105370728* |  |
| *LOC105378536 - LOC107984184* |  |
| *ATXN2* |  |
| *GABBR2* |  |
| *RPS3AP9 - CLPTM1LP1* |  |
| *LINGO2* |  |
| *LOC107986582 - BTN3A2* |  |
| *CLN8* |  |
| *LOC105375694 - RPL23P9* |  |
| *ABHD17C* |  |
| *ZNF536* |  |
| *ZDHHC2* |  |
| *CAND1.11* |  |
| *LOC105375856* |  |
| *KCNQ1* |  |
| *FGF5* |  |
| *RPL23AP96 - DEFA6* |  |
| *LINC00327* |  |
| *LOC105376658 - DKFZp779M0652* |  |
| *MCPH1* |  |
| *LOC105370791* |  |
| *PRKCE, LOC102724965* |  |
| *TEX41* |  |
| *HAUS8* |  |
| *DBH, DBH-AS1* |  |
| *CELA2A* |  |
| *LOC105371811 - LOC105371812* |  |
| *DNAAF1* |  |
| *CCL20 - TDGF1P2* |  |
| *LARGE - LOC105373010* |  |
| *APOH - RNA5SP444* |  |
| *SETD7* |  |
| *SFRP5 - LOC107984260* |  |
| *LRP1B* |  |
| *TRAPPC9* |  |
| *LOC107984630* |  |
| *ZSWIM7* |  |
| *CDH19* |  |
| *LOC105372221 - LOC105372224* |  |
| *OGDH* |  |
| *DPPA3P2* |  |
| *LOC100418832 - LOC100418834* |  |
| *COL4A2* |  |
| *RAPSN* |  |
| *LOC101927697 - EBF1* |  |
| *LOC105369166* |  |
| *XRCC4* |  |
| *STK39* |  |
| *GOSR2* |  |
| *ZNF652* |  |
| *CCDC141* |  |
| *KERA - LUM* |  |
| *LINC01249 - RNU6-649P* |  |
| *CACNA2D4* |  |
| *LOC102724863 - NEK4P3* |  |
| *OR9Q1* |  |
| *MRPL23 - IGF2* |  |
| *CYP27A1* |  |
| *HSF2BP* |  |
| *HCG24 - COL11A2* |  |
| *SNX16* |  |
| *TRPC4* |  |
| *TAP2* |  |
| *CYB561 - LOC342541* |  |
| *SWAP70* |  |
| *LOC105369687 - LOC105369688* |  |
| *LOC105369743* |  |
| *KLHL29* |  |
| *LOC105370003* |  |
| *MECOM* |  |
| *TBC1D7-LOC100130357, PHACTR1* |  |
| *RNU6-783P - LOC107984378* |  |
| *WFDC1* |  |
| *HECTD4* |  |
| *ANLN* |  |
| *ITGA11* |  |
| *LOC105375975 - LOC101929446* |  |
| *CNNM2* |  |
| *TCEA2* |  |
| *LINC00240 - VN1R12P* |  |
| *LOC105379082* |  |
| *SLMO2P3 - MIR633* |  |
| *RNU1-146P - LOC105369682* |  |
| *RPS29P9 - LOC102724714* |  |
| *LOC105379231* |  |
| *LOC105377992 - LOC105377989* |  |
| *CERS5* |  |
| *PALLD* |  |
| *BDNF* |  |

^†^, candidate genes replicated in GWAS at a genome-wide significant threshold (P <10^-7^)

**References:**

1. Rossier BC, Bochud M, Devuyst O. The hypertension pandemic: An evolutionary perspective. Physiology. 2017;32:112–25.

2. Sanada H, JE J, PA J. Genetics of Salt-Sensitive Hypertension. Curr Hypertens Rep. 2011;13:55–66.

3. Mattson DL, Liang M. Hypertension: From GWAS to functional genomics-based precision medicine. Nat. Rev. Nephrol. 2017; 10.1038/nrneph.2017.21

4. Padmanabhan S, Caulfield M, Dominiczak AF. Genetic and Molecular Aspects of Hypertension. Circ. Res. 2015;116:937–59.

5. Warren HR, Evangelou E, Cabrera CP, Gao H, Ren M, Mifsud B, et al. Genome-wide association analysis identifies novel blood pressure loci and offers biological insights into cardiovascular risk. Nat. Genet. 2017; 10.1038/ng.3768

6. Padmanabhan S, Newton-Cheh C, Dominiczak AF. Genetic basis of blood pressure and hypertension. Trends Genet. 2012;28:397–408.

7. Padmanabhan S, Joe B. Towards Precision Medicine for Hypertension: A Review of Genomic, Epigenomic, and Microbiomic Effects on Blood Pressure in Experimental Rat Models and Humans. Physiol. Rev. 2017;97:1469–528.

8. (GWAS Catalogue: <https://www.ebi.ac.uk/gwas/>)
